# Experimenting with the High Potencies

**Published:** 1884-04

**Authors:** R. O. Wood

**Affiliations:** North Weare, N. H.


### EXPERIMENTING WITH THE HIGH POTENCIES, WITH TWO CASES FOR CONSULTATION.

*E. J. Lee, M. D.*

DEAR SIR:—Some months ago I resolved to investigate the high potency theory. Was told that you were a disciple thereof and published a medical journal advocating its principles and practice. I was then taking two allopathic journals, two homœopathic, and one eclectic, and am yet. You kindly sent me a few numbers of your journal for examination; but finding everything so radically different from all others I had read, I passed them over to others whom I thought would better appreciate your doctrine of "infinitesimal nothingness," as I have been wont to call it. Through the constant advice of my friend, Dr. C——, of H——, N. H. (who, as he says, was converted to the high potency theory *accidentally*, as I have been), I bought some Ars.$^{30}$ and Bry.$^{30}$ and thought I would test its efficacy the first opportunity. The very next day after receiving them I found a patient very much emaciated, who complained of feeling tired, weak, and desired to lie down on the bed or sofa continually. She complained also of constant thirst, but only wanted a "*swallow of water at a time, but often.*" "Now Arsenic$^{30}$," said I to myself; "I will try it, any way, but I will leave the same remedy in third in another glass, with directions to use goblet No. 2 if goblet No. 1 does not benefit her." In goblet No. 1 I put in ten drops of Ars.$^{30}$ and filled it half full of water, with instructions to take a teaspoonful twice daily; but I also directed that in case no perceptible effect was seen in twelve hours, to drop No. 1 and use from No. 2 goblet.

The next morning I called to see the result, and, on entering the house, the daughter saluted me with—

"Why, Doctor, what have you been giving my mother? I gave her a teaspoonful of No. 1 and within an hour she got off from the bed and walked all over the room, went to the cupboard, and got out her knitting-work. Her eyes snapped and she talked a blue streak, and I did not dare to give her any more of No. 1."

I assured her that it could not possibly be the effect of the medicine, and proceeded to inform her and the family how very thin (?) the remedy was that I had given her and how it had been prepared. She insisted that she knew it *was the effect of the medicine* and that she should not dare to give her mother any more of it. I finally induced her to "try again," and I would watch its effect and see if it could possibly be the medicine.

At two o'clock I gave her another teaspoonful and within an hour she repeated her "antics" of the day before, much to my surprise and gratification. Three-fourths of the remaining contents of the goblet was now poured out and again filled with water, and even then a teaspoonful had more than the desired effect.

I am now about in the same position as a young sister on the "anxious seat" in a Free Methodist Church, and "*almost persuaded*" to declare myself a convert to the high potency theory. If I am still in darkness I pray for "*more light*," and, once obtaining it, I will as freely give as I receive it, and I will fight as zealously to disseminate its truth and light as I have in the past opposed it. I have a few cases on hand which would afford good tests for the efficacy of high potencies.

Case I.—A lady, aged thirty, dark hair and eyes, medium physique—a bright brunette—flushed cheeks, and in appearance the picture of health. She informs me she had inflammation of lungs at thirteen, followed by bloating for years, and which still exists. Menstruation at fourteen. Coxalgia, with soreness of spine above. Palpitation at times early in the morning. Prolapsus uteri at fifteen. Retention of urine, with frequent and ineffectual desire and with scalding and smarting. Pain in back opposite left ovary, attended by trouble in the stomach, with a gnawing soreness and full feeling, with nausea sometimes. Spinal irritation and a violent, irritable temper; at times melancholy, but naturally is cheerful and happy. Pain in back of head, affecting eyes. Using eyes when they are feeling badly affects the spine. Eyes at times feel as though they were full of sticks, and, when steadily and persistently looking at an object, feel as though they were being pressed out of the head. Light affects them unfavorably. The present symptoms now principally are: *Pain in left ovarian region and back opposite*, with spinal irritation. The *head is easily confused.* Using eyes much causes severe headache.

Case II.—A man about sixty years of age with hypertrophy of heart; has charge of a gang of hands in a gas-fitting shop, losing no time. His nature is very sociable, but excitable and easily frightened. Severe constipation exists, and, most prominently, the following symptoms: Sleeplessness in the after part of the night, and while sleeping there is profuse *cold* sweat, alternating with "goose flesh" (as the wife called it) so rough as to almost scratch his wife's hand. His wife will awaken him and ask him if he is cold. He replies: "No, I am warm and comfortable." The last-named symptoms are *sure* to follow a social glass with one of his employees whom he finds in the evening, though he does not indulge very often, even with a friend. He recently applied to a "regular" (allopath) for a remedy for the relief of his constipation and was ordered to take four cathartic pills daily, and at the same time admonished to be very careful of himself or he would fall a victim to epilepsy. This has so worried him that he is very despondent and frequently assures his wife that he shall soon have epilepsy and die; is very melancholy and at times excited and weeping.

If some of the kind readers of your journal will send me remedies to apply in the above cases and will also do me the especial favor to mail me a few *reliable very high potencies* of remedies most in use in practice, I will preserve and use the same for the benefit of suffering humanity, and in return will not only give them my unfeigned gratitude, but will withal remit the amount of their bill for same and in turn will render a like favor to any or all who may apply to me for a like favor.

Fraternally, etc.,

R. O. Wood, M. D.

*North Weare, N. H.*

P. S.—Where can I obtain a copy of Hahnemann's *Chronic Diseases?*

